# Evaluation of biological sex on endstage pathobiology and regenerative treatment of volumetric muscle loss

**DOI:** 10.1038/s41598-025-05166-y

**Published:** 2025-07-01

**Authors:** Jessica M. Motherwell, Isabella J. Meerzaman, Sergey S. Kanovka, Michael S. Valerio, Claudia E. Hernandez, Zachary G. Davis, Andrew R. Clark, Stephen M. Goldman, Christopher L. Dearth

**Affiliations:** 1 Extremity Trauma and Amputation Center of Excellence, Defense Health Agency, Falls Church, VA USA; 2 Department of Surgery, Uniformed Services University of the Health Sciences, Bethesda, MD USA; 3 C2 Alaska LLC, San Antonio, TX 78249 USA; 4 The Henry M. Jackson Foundation for the Advancement of Military Medicine, Bethesda, MD USA

**Keywords:** Sex characteristics, Wound healing, Skeletal muscle, Regeneration, Trauma, Musculoskeletal models, Regenerative medicine

## Abstract

Volumetric muscle loss (VML) is a severe injury resulting in substantial skeletal muscle loss, leading to a complex pathology that culminates in suboptimal tissue repair and significant long-term functional deficits. This study employs a rodent model of VML to investigate the impact of biological sex on the injury pathobiology and its potential influence on the response to autologous minced muscle grafting (MMG) as a regenerative therapy. While no significant differences were observed between the end-stage male and female responses to VML, both male and female subjects appeared to benefit from MMG treatment. Females demonstrated improved neuromuscular function, while males exhibited reduced fibrosis at the site of injury. Additional differences in the wound healing response included distinct variations in myofiber characteristics, with females exhibiting a lower proportion of Type 2a fibers and elevated levels of myogenin. These findings suggest that regenerative therapies, such as MMG, may exhibit sex-specific benefits. Future studies will further explore sexual dimorphism in the acute response to injury to identify potential therapeutic targets that may yield greater therapeutic efficacy for each sex.

**Supplementary Information:**

The online version contains supplementary material available at 10.1038/s41598-025-05166-y.

## Introduction

Volumetric muscle loss (VML) is a substantial and irrecoverable injury affecting both military and civilian populations that is characterized by extensive loss of skeletal muscle beyond the endogenous capabilities for tissue repair^1,2^. The outcomes of such injuries is severe scarring, considerable long-term functional deficits, and a diminished quality of life for affected individuals^3^. Conventional clinical practice for the management of VML involves the use of autologous muscle flaps^4–6^. While this approach aims to reduce complications related to traumatic injury, it often results in secondary donor site morbidity and fails to promote the restoration of end-organ function^7^. Such outcomes contribute to patients requesting delayed amputations in lieu of living with chronic functional deficits^8^. Given the profound impact of VML on quality of life and the limitations of current treatment strategies, it is essential to examine critical factors that could influence wound healing and functional recovery, including potential sex-based differences.

In the past decade, significant progress has been made towards understanding the innate pathobiology of VML injuries and the consequent disruption of skeletal muscle repair. Research indicates that the tissue responds to the injury with a heightened and prolonged inflammatory response. Studies have shown that this dysregulated inflammatory response coupled with inadequate debris clearance, influences the proliferation and differentiation of fibro-adipogenic progenitor cells into fibroblastic and adipogenic phenotypes. These cells then promote extensive fibrotic and adipose tissue accumulation^9,10^. While this response presents as a compensatory repair mechanism, it hinders satellite cell-mediated *de novo* myogenesis, thereby limiting the potential for a robust endogenous skeletal muscle regeneration.

It is important to note that the extant literature that elucidated these effects predominantly used male subjects. Furthermore, few studies have explicitly investigated sex as a biological variable with respect to the pathobiology of VML, and no study has directly investigated its impact on the efficacy of regenerative therapies. It is crucial to consider sex-based differences in VML, as evidence suggests that males and females may exhibit distinct inflammatory and regenerative responses following injury^11,12^. Understanding these differences could lead to more tailored and effective therapeutic strategies that improve definitive outcomes for both sexes following this traumatic injury.

Preclinical studies have evaluated sex-based differences using recoverable skeletal muscle injury models to examine the influence of biological sex on the regeneration process^13–17^. One such study demonstrated that female mice recover muscle fiber size and function more rapidly than males, with these advantages persisting long-term^13^. Another study demonstrated that estrogen was essential for the maintenance and differentiation of satellite cells, the cells responsible for repairing skeletal muscle, following injury^15^. Furthermore, evidence suggests that estrogen regulates the satellite cell compartment in females by sustaining the number of cells in the muscle, and the loss of specific estrogen receptors results in impaired muscle strength following injury^14^. However, given the irrecoverable nature of skeletal muscle damage following VML, it is critical to understand the influence of physiological differences between the sexes on injury pathobiology and response to regenerative treatment. By elucidating these differences, therapeutic strategies can be tailored by leveraging this knowledge, thereby facilitating improved functional recovery following VML.

Research in both large and small animal models has demonstrated that autologous minced muscle graft (MMG) is a promising regenerative intervention for VML^18–24^. This method provides essential cellular and structural components, such as satellite cells and basal lamina, which are critical for muscle fiber regeneration and restoration of function^25–27^. However, existing studies have primarily focused on either male or female animal models to evaluate the efficacy of MMG treatment, without direct comparison to assess the influence of sex-based differences^9,18,23,28^.

In the present study, we aim to address this knowledge gap using a well-established rodent model of VML injury. With MMG as a model regenerative treatment, we evaluated sex-based differences on definitive outcomes and muscle functional recovery following traumatic injury in intact male and female rats.

## Results

### Sex differences in body and muscle weight following VML injury and MMG treatment

Body weight and muscle mass from both VML-injured and contralateral limbs were evaluated for differences by biological sex or regenerative treatment (Table 1). Body weights at pre- and post-injury timepoints varied by sex, where males on average weighed more than females. Additionally, weight gain as a percentage of initial body mass was different by sex and treatment, where females gained less than their male counterparts for the duration of the study (*P* < 0.0001). Tibialis anterior (TA) and extensor digitorum longus (EDL) muscles in both injured and contralateral uninjured limbs weighed less in females than in males, with no differences by regenerative treatment. Analysis of the ratio between VML and contralateral TA muscles revealed a significant interaction between sex and treatment (*P* = 0.0294), with untreated females showing a lower ratio compared to all other groups. However, when muscle mass was normalized to body weight, no differences by sex or MMG treatment was observed in the VML or uninjured muscles, with the exception of the contralateral TA (*P* < 0.0001). Regenerative treatment had no effect on muscle mass in VML injured limbs for either males or females compared to untreated.

**Table 1:** Body weights and muscle Mass.

			Male		Female	
		n	**No Repair**	**Minced Graft**	**No Repair**	**Minced Graft**
Body Weight						
Pre-Injury (g)	a	18	343 ± 7	345 ± 9	245 ± 20	243 ± 10
56-Days Post (g)	a	18	423 ± 25	433 ± 19	278 ± 23	286 ± 14
Wt Gain (% Initial)	a, b	18	23.6 ± 6.6	25.5 ± 5.3	13.8 ± 4.6	17.8 ± 7.0
Muscle Mass, g						
VML – TA	a	17–18	0.697 ± 0.115	0.696 ± 0.130	0.444 ± 0.139	0.531 ± 0.061
Contralateral – TA	a	18	0.858 ± 0.080	0.894 ± 0.061	0.616 ± 0.048	0.628 ± 0.052
VML – EDL	a	17–18	0.194 ± 0.034	0.198 ± 0.049	0.116 ± 0.025	0.131 ± 0.026
Contralateral – EDL	a	18	0.183 ± 0.023	0.190 ± 0.019	0.129 ± 0.016	0.127 ± 0.012
VML: Contralateral – TA	c	17–18	0.812 ± 0.102	0.779 ± 0.135	0.724 ± 0.211	0.844 ± 0.080^*^
Muscle Mass, g/kg BW						
VML – TA		17–18	1.649 ± 0.263	1.612 ± 0.297	1.604 ± 0.482	1.855 ± 0.201
Contralateral – TA	a	18	2.025 ± 0.137	2.067 ± 0.137	2.228 ± 0.178	2.195 ± 0.161
VML – EDL		17–18	0.458 ± 0.081	0.456 ± 0.112	0.419 ± 0.096	0.457 ± 0.093
Contralateral – EDL		18	0.430 ± 0.041	0.439 ± 0.037	0.465 ± 0.063	0.443 ± 0.041
VML: Contralateral – TA		17–18	0.812 ± 0.102	0.779 ± 0.135	0.724 ± 0.211	0.816 ± 0.144

Data are presented as mean ± SD

Statistical significance (*P* < 0.05) was determined by two-way ANOVA with Sidak’s post hoc test

a = main effect by biological sex; b = main effect by treatment; c = interaction; *difference between NR and MG within sex

Wt = weight; TA = tibialis anterior; EDL = extensor digitorum longus; BW = body weight; NR = no repair; MG = minced graft

### Evaluation of neuromuscular function following VML injury

**Fig. 1:**
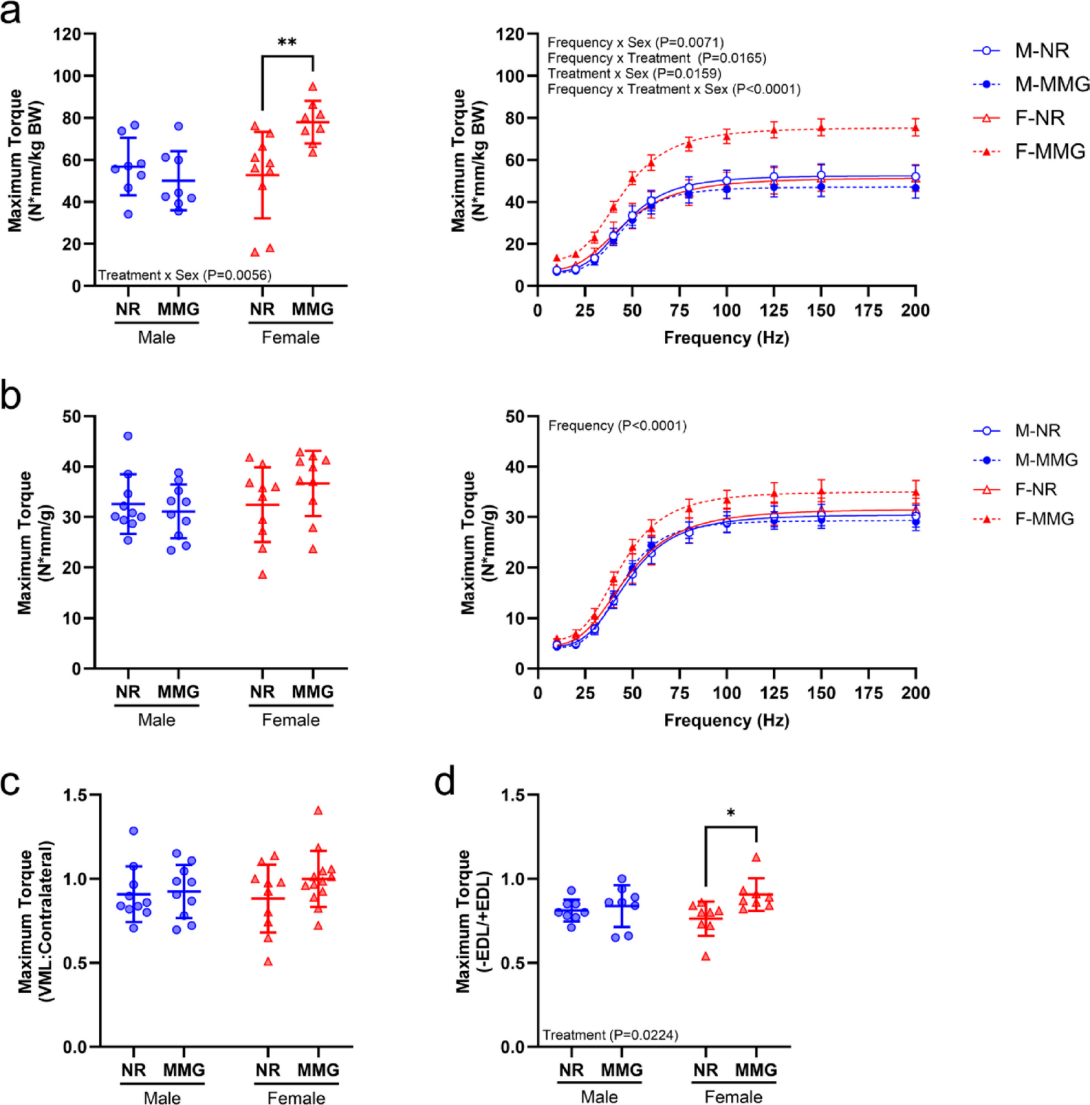
Neural-evoked functional assessments from VML-injured muscles at 56 days. Average maximum torque values were normalized to body weight (**a**) or muscle weight (**b**) to evaluate the effects of biological sex and treatment on muscle function post-VML (*n* = 8–10 muscles/group). Maximum torque values were recorded at increasing frequencies and normalized to body weight (*n* = 8–10 muscles/group). (**c**) Maximum isometric torque values from VML-injured muscles were normalized to the uninjured contralateral values to assess recovery of muscle function (*n* = 10–13 muscles/group). (**d**) Maximum isometric torque values post-tenotomy of the EDL were normalized to pre-tenotomy values to examine the effects of biological sex and treatment on force imbalance due to VML (*n* = 8 muscles/group). Females and males are represented by red and blue markers, respectively. Data are presented as mean ± SD. Statistical significance is indicated by *(*P* < 0.05) and **(*P* < 0.01), as determined by two-way or three-way ANOVA with Sidak’s post hoc test. NR = no repair; MMG = minced muscle graft; BW = body weight; EDL = extensor digitorum longus.

Neuromuscular function was assessed in VML-injured limbs at 56 days post-injury. Maximum isometric torque values normalized to body weights revealed a significant interaction between sex and treatment (*P* = 0.0056), with females receiving MMG showing increased muscle function compared to untreated females (Fig. 1a). Specifically, the Female-MMG group exhibited a 32.3% increase in maximum torque compared to the Female-NR group (*P* = 0.0036). Further analysis of isometric torque at varying frequencies demonstrated a significant interaction among frequency, sex, and treatment, with MMG-treated females consistently displaying higher torque across frequencies compared to all other groups (Fig. 1a). When isometric torque values were normalized to muscle mass, no differences were observed by sex or treatment across the full range of stimulation frequencies (Fig. 1b). The VML-to-contralateral ratio of maximum torque values showed no differences by sex or treatment (Fig. 1c). Additionally, post-EDL tenotomy maximum isometric torque, normalized to pre-tenotomy values, indicated that MMG treatment alleviated the force imbalance caused by VML injury (*P* = 0.0224) (Fig. 1d). Untreated females showed more imbalance compared to MMG-treated (*P* = 0.0146), whereas no differences were observed between the male groups (*P* = 0.8413).

### Fibrotic deposition is reduced in males treated with MMG

**Fig. 2:**
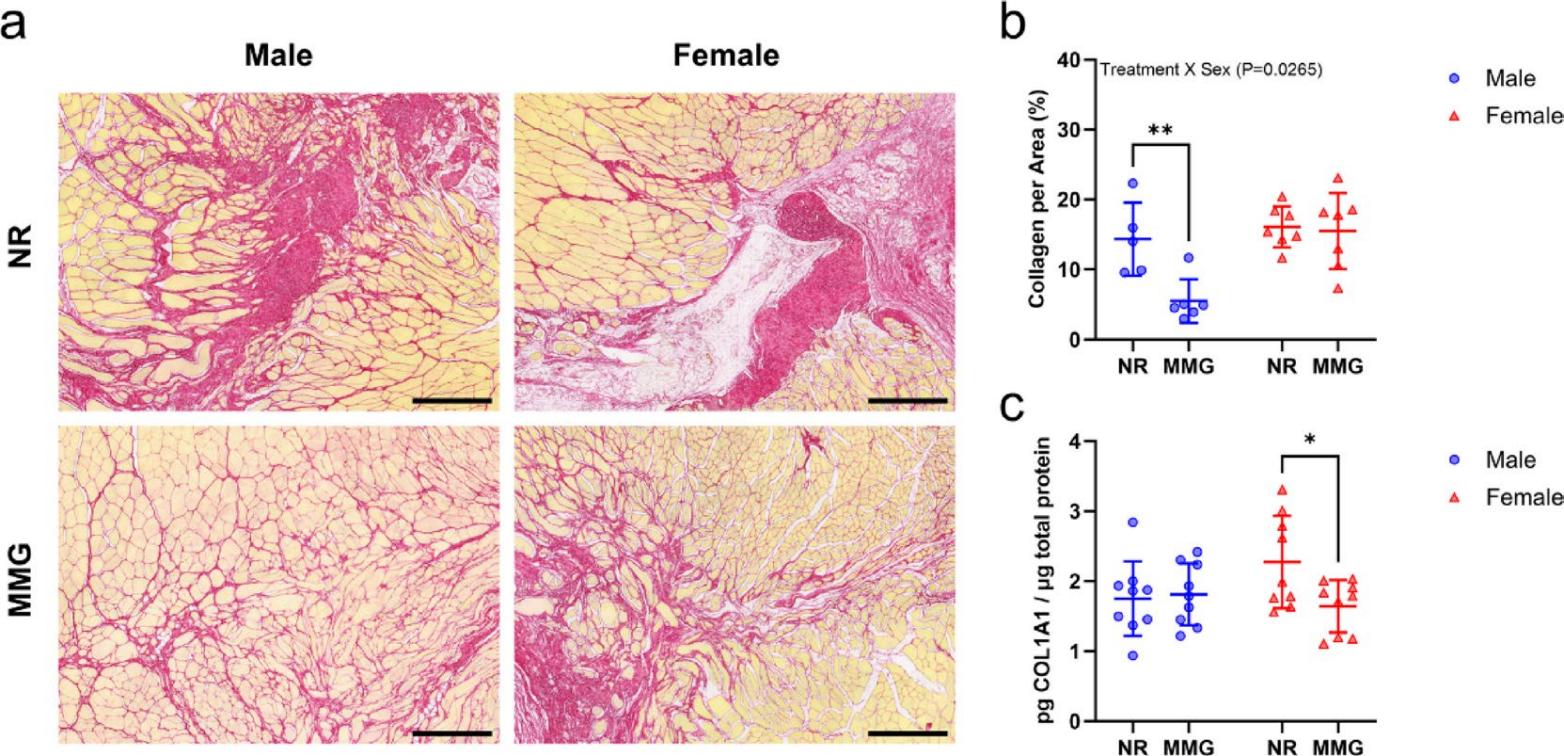
Assessment of collagen deposition at the VML defect site. (**a**) Representative picrosirius red-stained muscle cross-sections at the defect site 56 days post-injury, with red indicating collagen and yellow identifying muscle fibers. Scale bars = 200 μm. (**b**) Quantitative analysis of collagen deposition in VML-injured muscles (*n* = 5–7 muscles/group). (**c**) Quantification of COL1 A1 protein levels in VML-injured muscles (*n* = 8–9 muscles/group). Females and males are represented by red and blue markers, respectively. Data are shown as mean ± SD. Statistical significance is denoted by *(*P* < 0.05) and **(*P* < 0.01), determined by two-way ANOVA with Sidak’s post hoc test. NR = no repair; MMG = minced muscle graft.

Muscle fibrosis resulting from VML injury was assessed for effects by biological sex or regenerative treatment. Figure 2a shows representative picrosirius red-stained tissues, highlighting significant fibrosis in all VML injuries except the male MMG-treated group. Quantitative analysis of collagen deposition (Fig. 2b) revealed a significant interaction between sex and treatment (*P* = 0.0265), indicating that males treated with MMG had markedly lower fibrosis compared to all other groups, with a 62% decrease compared to untreated males (*P* = 0.0052). Untreated females showed increased COL1 A1 protein expression, a key marker of scar tissue formation, compared to MMG-treated females (*P* = 0.0271). However, we found no differences attributed to biological sex (*P* = 0.3125) or regenerative treatment (*P* = 0.1045) (Fig. 2c).

### Assessment of fast twitch fibers and myogenic markers after VML injury

**Fig. 3:**
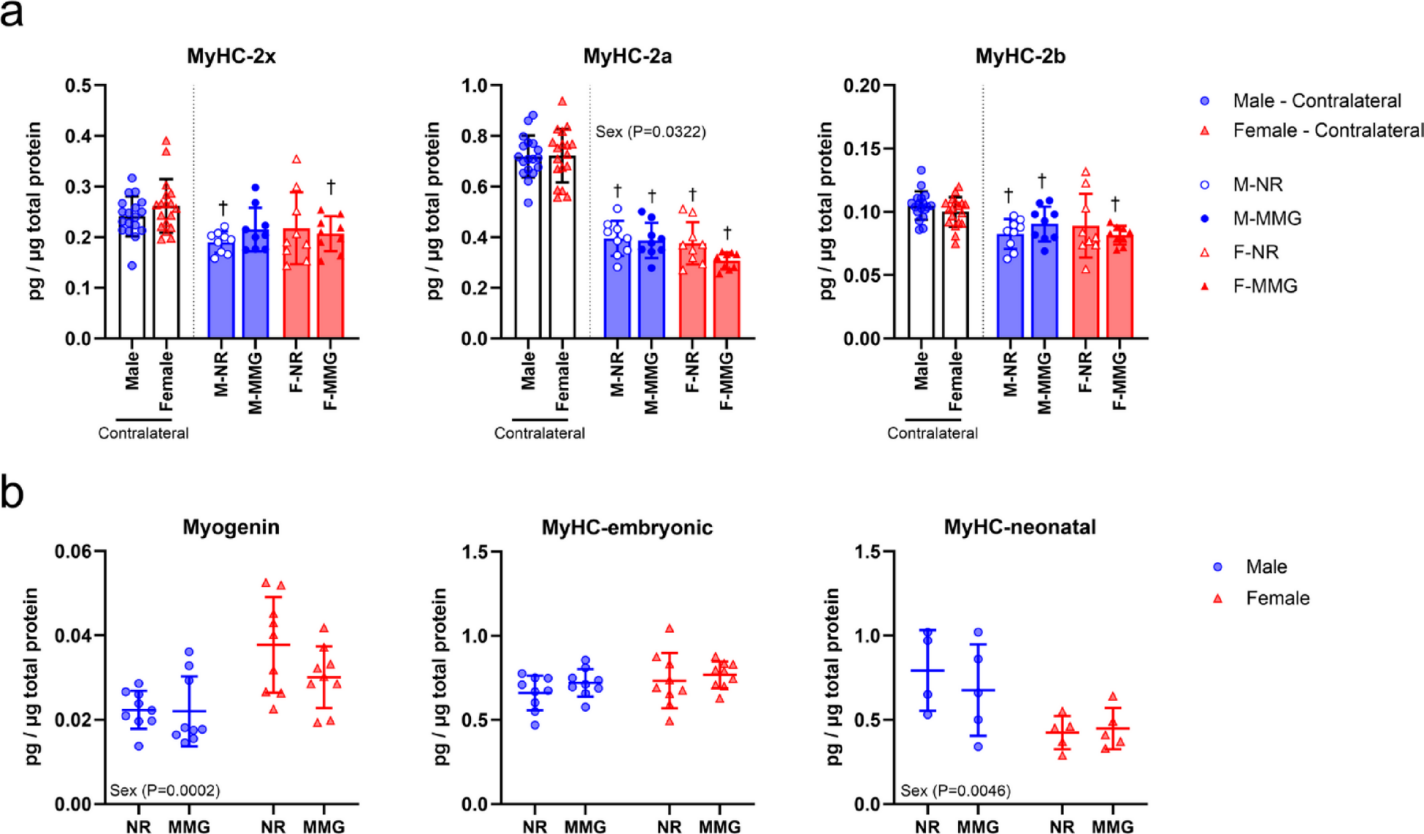
Evaluation of MyHC and pro-myogenic protein expression in VML-injured muscles. (**a**) Protein expression in VML-injured and uninjured contralateral limbs were quantified for Type 2x (MyHC-2x), Type 2a (MyHC-2a), and Type 2b (MyHC-2b) fibers at 56 days post-injury. To evaluate the effects of biological sex and regenerative treatment on muscle fiber composition following VML, protein levels for each fast twitch fiber type were compared between injured limbs (*n* = 8–9 muscles/group). To evaluate changes in muscle fiber composition post-injury, protein levels between VML-injured and uninjured contralateral limbs were compared (*n* = 7–9 per group). (**b**) Protein markers indicative of muscle regeneration were evaluated from VML-injured muscles (*n* = 3–9 muscles/group). All protein values were normalized to total protein content prior to analysis. Females and males are represented by red and blue markers, respectively. Data are shown as mean ± SD. Statistical comparisons between VML-injured limbs was determined by two-way ANOVA (*P* < 0.05). Statistical comparisons between VML-injured and respective contralateral limbs was determined by Student’s T-test or non-parametric Mann-Whitney test (*P* < 0.05). NR = no repair; MMG = minced muscle graft.

Fast twitch muscle fibers were evaluated in VML and contralateral limbs by measuring myosin heavy chain (MyHC) isoforms associated with fiber Type 2x (MyHC-2x), Type 2a (MyHC-2a), and Type 2b (MyHC-2b) at 56 days post-injury (Fig. 3a). Males demonstrated an increase in Type 2a fibers in VML-injured limbs compared to females (*P* = 0.0322), although no differences were observed by regenerative treatment. Conversely, no treatment- or sex-related differences were observed for Type 2x and 2b fibers. When compared to uninjured contralateral muscles, a general decrease in MyHC expression levels was observed, irrespective of biological sex or treatment. Protein markers associated with myocyte commitment (myogenin) and nascent myotube formation (embryonic and neonatal myosin heavy chain) were evaluated at the 56-day study endpoint (Fig. 3b). Our results revealed differences in myogenin levels by sex (*P* = 0.0002), with females exhibiting a 35% increase compared to males. In contrast, analysis of embryonic myosin heavy chain (MyHC-embryonic) protein levels indicated no differences by sex nor treatment. Interestingly, neonatal myosin heavy chain (MyHC-neonatal) showed difference by sex (*P* = 0.0046), where males overall showed an increase in expression compared to females. No differences attributed to regenerative treatment were observed across all three pro-myogenic markers evaluated.

### Myofiber distribution and quantification in VML-Injured muscles

**Fig. 4:**
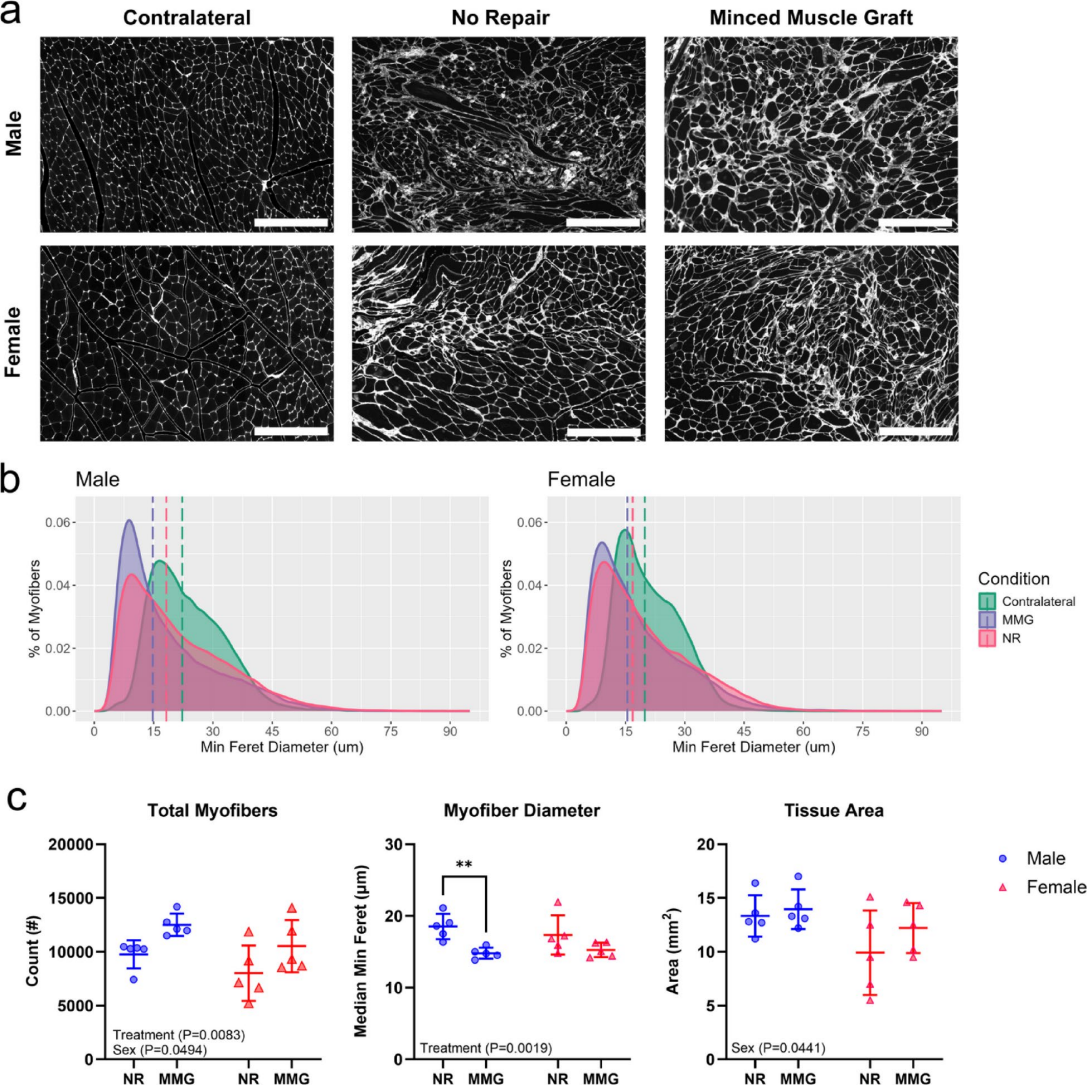
Analysis of myofiber morphology and regenerative outcomes following VML injury. VML-injured and uninjured contralateral muscle cross-sections were stained with wheat germ agglutinin (WGA) to visualize individual myofibers for analysis of measurement parameters. (**a**) Representative images from the injury site (or corresponding location in uninjured muscles) are shown for each experimental group, from muscles harvested at 56 days post-injury. Scale bars = 200 μm. (**b**) Myofiber distributions were stratified by minimum Feret diameter, derived from *n* = 5 muscle cross-sections for VML-injured groups and *n* = 6 for uninjured. The dashed line indicates the median myofiber minimum Feret diameter for each group. Statistical significance was determined using Kruskal-Wallis test with Dunn’s post hoc test. (**c**) Total muscle fiber count, median myofiber diameter, and tissue area were quantified to assess the effects of biological sex and regenerative treatment on muscle regeneration following VML (*n* = 5 muscles/group). Data are presented as mean ± SD. Statistical significance is indicated by **(*P* < 0.01), assessed using two-way ANOVA with Sidak’s post hoc test. NR = no repair; MMG = minced muscle graft.

To investigate muscle healing outcomes post-VML, we stained muscle cross-sections for wheat germ agglutinin and assessed myofiber measurement parameters for regeneration (Fig. 4). Representative images of muscle cross-sections at the defect site are shown in Fig. 4a. Analysis of myofiber distributions stratified by minimum Feret diameter revealed differences between untreated and MMG-treated males (*P* < 0.0001) and females (*P* < 0.0001), where treated muscles had a higher proportion of smaller myofibers (Fig. 4b). Quantification of the total number of myofibers in the cross-sections demonstrated significant effects by treatment (*P* = 0.0083) and biological sex (*P* = 0.0494). Treatment with MMG led to an increase in the absolute number of myofibers for both sexes, while females had fewer myofibers overall compared to males (Fig. 4c). Interestingly, males treated with MMG showed no differences in myofiber counts compared to uninjured contralateral muscles (*P* = 0.3232) (Supplemental Fig. S1). Comparing median myofiber diameters highlighted a significant effect by treatment (*P* = 0.0019), indicating that both males and females receiving the MMG intervention had smaller diameters than their untreated counterparts (Fig. 4c). Further evaluation of cross-sectional area of VML-injured muscles showed an effect by sex (*P* = 0.0441), where females had smaller tissue area compared to males, consistent with the muscle mass findings presented in Table 1.

### Sex differences and treatment effects on neuromuscular markers

**Fig. 5:**
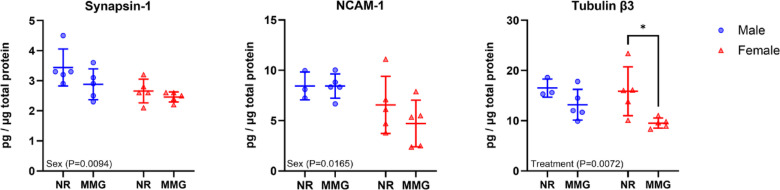
Quantification of neuromuscular protein markers in VML-injured muscles. Protein levels in VML-injured muscles were quantified for neuromuscular markers at 56 days post-injury (*n* = 3–5 muscles/group). All protein values were normalized to total protein content prior to analysis. Females and males are represented by red and blue markers, respectively. Data are shown as mean ± SD. Statistical significance is denoted by *(*P* < 0.05), determined by two-way ANOVA with Sidak’s post hoc test. NR = no repair; MMG = minced muscle graft.

Protein markers associated with neuromuscular synapses (synapsin-1 and neural cell adhesion molecule [NCAM-1]) and axons (tubulin β3) were evaluated at the study endpoint (Fig. 5). The data showed that biological sex effects protein expression of synapsin-1 (*P* = 0.0094) and NCAM-1 (*P* = 0.0165), where males had increased levels compared to females. Interestingly, MMG-treated groups had reduced tubulin β3 levels (*P* = 0.0072) in both males and females compared to untreated.

### Analysis of intramuscular adipose tissue formation post-VML

**Fig. 6:**
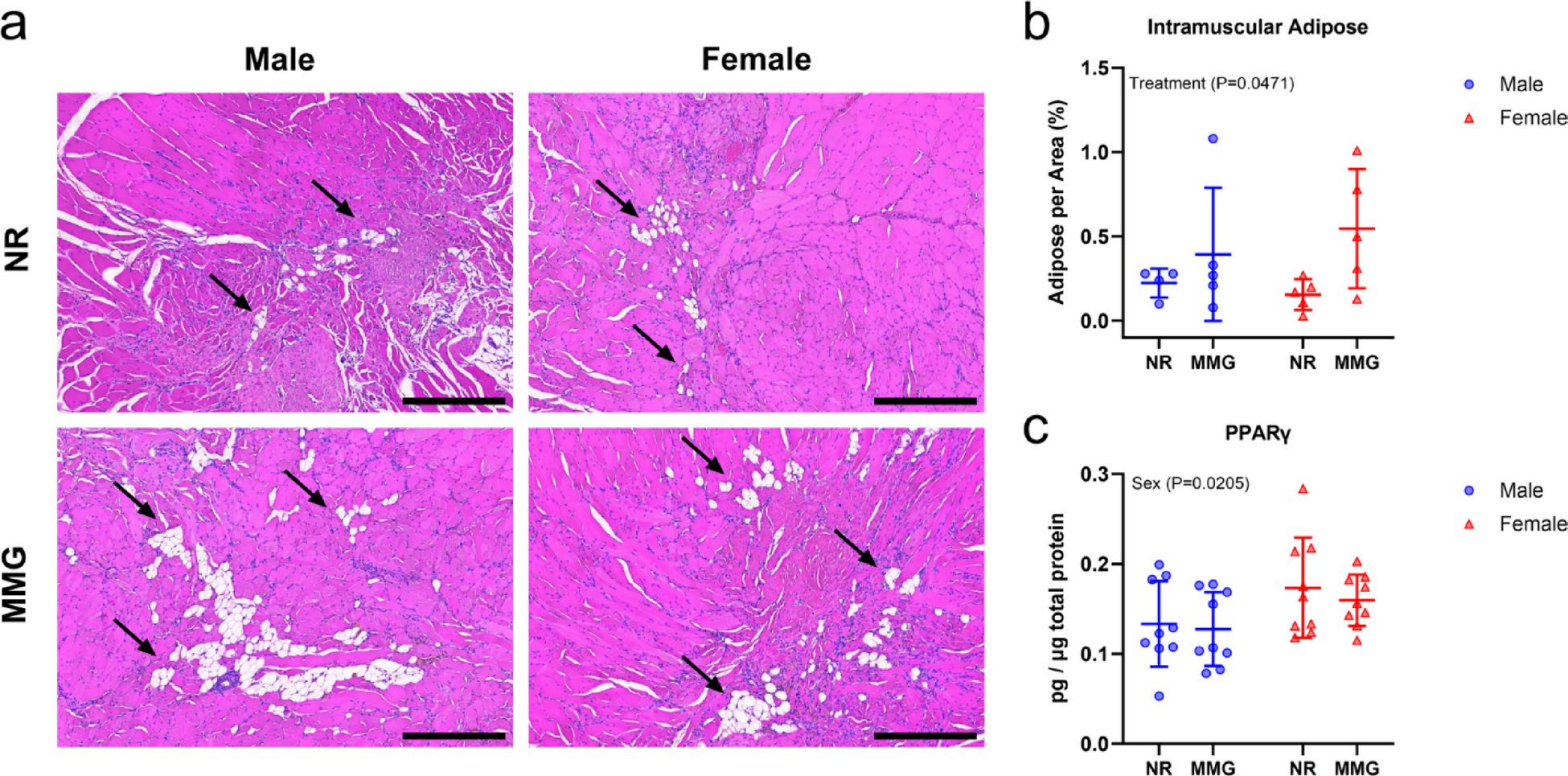
Assessment of intramuscular adipose deposition at the VML defect site. (**a**) Representative H&E stained muscle cross-sections at the defect by 56 days-post injury. Adipose deposits are indicated by the black arrows. Scale bars = 200 μm. (**b**) Quantification of intramuscular adipose deposition in VML-injured muscles (*n* = 4–5 muscles/group). (**c**) Quantification of PPARɣ protein levels in VML-injured muscles (*n* = 8–9 muscles/group). Females and males are represented by red and blue markers, respectively. Data are shown as mean ± SD. Statistical significance was determined by two-way ANOVA (*P* < 0.05). NR = no repair; MMG = minced muscle graft.

Intramuscular adipose tissue formation was investigated in VML-injured muscles at 56 days post-injury. Representative micrographs of the VML defect site for each experimental group are shown in Fig. 6a. Quantitative analysis of adipose tissue, normalized by total muscle cross-sectional area (Fig. 6b), showed differences in accumulation based on treatment (*P* = 0.0471). In particular, animals that received MMG had increased intramuscular adipose at the VML defect site compared to untreated animals. To further explore the molecular mechanisms underlying adipose formation, the protein expression of peroxisome proliferator-activated receptor-gamma (PPARγ) was examined (Fig. 6c). This analysis revealed a significant difference by biological sex (*P* = 0.0205), with females exhibiting higher protein levels compared to males. No observable differences linked to MMG treatment were detected (*P* = 0.5166).

## Discussion

The objective of this study was to determine whether biological sex influences the long-term pathobiology of VML and the response to regenerative therapy in a rodent model. Our results show that both sexes experienced improved muscle repair following MMG treatment, with females demonstrating increased muscle function and males exhibiting a reduction in fibrotic deposition, a key factor that hinders healthy muscle regeneration. These findings suggest that while biological sex may influence specific aspects of VML pathology, MMG treatment benefits both males and females.

The results from this study provide insights into the relationship between biological sex and regenerative treatment on muscle repair following VML injury. Notably, females treated with autologous muscle grafts demonstrated improved neural-evoked muscle function at 56 days post-injury, exhibiting a 32.3% increase compared to untreated females. However, this improvement was less pronounced when normalized to muscle mass (Fig. 1b), suggesting that while MMG treatment can mitigate functional deficits associated with VML, it may not fully restore muscle mass. In contrast, there were no treatment-related differences in VML-injured muscle weights, indicating that while functional improvements were observed in the female group, additional pro-myogenic strategies may be required for complete muscle mass restoration beyond the use of autologous grafts. Neuromuscular protein markers associated with synaptic function and axonal sprouting were also evaluated in VML-injured muscles at the study endpoint^29–34^. Our findings demonstrated that biological sex impacts the expression of synapsin-1 and NCAM-1, with males exhibiting higher protein expression than females at 56 days post-injury. Additionally, MMG treatment reduced tubulin β3 protein levels in both males and females, indicating a treatment effect on axonal protein expression. Overall, these findings highlight sex-specific responses to regenerative treatment following VML injury, suggesting that while autologous muscle grafts offer modest functional improvements, further approaches are required to fully restore muscle mass and neuromuscular function in both sexes.

We evaluated myofiber morphology to determine whether biological sex or MMG treatment impacted long-term muscle regeneration outcomes at the study endpoint. The results demonstrated that MMG treatment improves muscle repair in both males and females following VML injury. Notably, we observed significant differences in myofiber distributions, with both males and females treated with MMG showing a higher proportion of smaller myofibers compared to their untreated counterparts, highlighting the effectiveness of MMG in promoting skeletal muscle regeneration. Interestingly, no differences in myofiber distributions were found between MMG-treated males and females (*P* = 0.0525). These findings suggest that biological sex does not significantly affect muscle regeneration in terms of myofiber morphology, while MMG treatment is beneficial across both sexes.

To further investigate the mechanisms underlying long-term muscle regeneration outcomes, we assessed protein markers associated with myocyte commitment and myotube formation. Our results revealed an increase in myogenin levels in females, while males showed an increase in neonatal myosin heavy chain levels. These findings suggest a possible temporal shift in muscle remodeling responses between the sexes, though additional studies are needed to examine myogenesis at earlier time points following VML injury. We also examined whether biological sex impacts fast-twitch muscle fibers post-VML. Our data indicated that males had a higher proportion of Type 2a fibers compared to females, consistent with previous research showing that males generally possess a greater proportion of Type 2a fibers^35^. Furthermore, we found no differences in fast twitch MyHC isoform expression related to the regenerative treatment administered. This finding parallels the results reported by Johnson et al. (2023), who evaluated the efficacy of a biomimetic sponge in treating VML in female rats. Their study found no differences in the proportions of fiber types among treated, untreated, and control groups when assessing immunostained muscle cross-sections^36^. While rodent TA muscles are predominantly comprised of fast twitch Type 2x and Type 2b fibers, we found no differences attributed to biological sex or regenerative treatment at 56 days post-injury^37^.

Regarding fibrotic deposition, males receiving MMG treatment exhibited a reduction in collagen levels compared to all other experimental groups. This reduction is crucial, as excessive fibrosis can impede healthy muscle regeneration and functional recovery. Previous research by Corona et al. (2013) demonstrated that autologous muscle graft treatment combined with voluntary wheel running reduced extracellular matrix deposition among regenerating myofibers in the VML defect region in rats. Additionally, their study showed that MMG treatment without rehabilitation led to decreased transforming growth factor-beta (TGFβ) gene expression up to 16 weeks post-injury^18^. We found that untreated females exhibited higher COL1A1 protein expression—a major component of scar tissue—compared to MMG-treated females, with no differences observed in males. However, other collagen subtypes and key fibrotic regulators, such as TGFβ and connective tissue growth factor, warrant further investigation, particularly given the reduction in collagen deposition observed in histological sections of MMG-treated males. These findings suggest a potential sex-specific response in fibrotic deposition with MMG treatment, where males show a reduction in collagen deposition, while females do not.

While this study explored the effects of MMG treatment on VML recovery, it also investigated adipose tissue formation as a relevant factor in VML pathology. Our results demonstrated that treatment with autologous muscle grafts increased intramuscular adipose within the VML defect. Similarly, McHale et al. (2012) reported an increase in intramuscular adipose tissue in both intact male and female mice following cardiotoxin injury in the TA muscle, with this effect persisting for the duration of the study^16^. These findings are consistent with prior research that has shown that increased adipose accumulation occurs within regenerating skeletal muscle tissue^38–41^. In our study, while biological sex did not influence adipose tissue formation post-VML, regenerative treatment with MMG led to an increase in intramuscular adipose tissue. Notably, protein levels of PPARγ, a key regulator of adipogenesis, were higher in females compared to males at the study endpoint, suggesting a predisposition towards intramuscular adipose accumulation following injury^42^. Beyond its role in adipogenesis, PPARγ is known to possess anti-inflammatory properties that modulate the immune response, including the suppression of pro-inflammatory cytokines^43–45^. Given that prior research has demonstrated the influence of biological sex on immune responses, along with the elevated PPARγ protein expression observed in females, further investigation is needed to determine whether females exhibit sustained anti-inflammatory properties following VML injury. Furthermore, given that specific cell populations within the VML injury site were not investigated in this study, future research is warranted to determine which cell-types contributed to the intramuscular adipose deposition observed, particularly in the MMG-treated groups. Together these findings raise important questions regarding the long-term implications of intramuscular adipose accumulation and PPARγ protein expression on muscle regeneration and functional recovery following VML injury.

While these data suggest that biological sex and regenerative treatment influence certain aspects of VML injury, there remains much to be explored. Our analysis primarily focused on long-term wound healing outcomes and the role of biological sex on muscle repair up to 56 days post-injury. However, examining earlier time points is crucial to gain a more comprehensive understanding of the temporal dynamics of muscle regeneration, and whether biological sex plays a role in shaping these outcomes. Previous research indicates sex-specific variations in the inflammatory response^11,12^, which is critical given the pivotal role of inflammation in VML pathology. Given that inflammation plays a critical role in wound healing and fibrotic tissue formation, understanding these early differences is essential for developing effective regenerative treatment strategies. Furthermore, the impact of sex hormones on injury response and recovery constitutes a crucial aspect of research into sexual dimorphism. This study did not incorporate hormone manipulation (i.e. estrogen modulation) as our primary aim was to investigate the influence of biological sex on long-term outcomes post-VML in intact males and females. Prior studies have explored sex differences in healthy skeletal muscle and the regenerative process following injury using intact male and females^13,46^. For example, You et al. (2023) demonstrated that female mice exhibit a more rapid recovery of muscle force and myofiber size compared to their male counterparts after barium chloride-induced muscle injury^13^. These findings underscore sex-based differences in muscle regeneration; however, our study specifically aimed to investigate the role of biological sex in the response to regenerative treatments and definitive outcomes following an irrecoverable injury model. Consequently, we did not explore the influence of biological sex at earlier post-injury time points. Future research will be necessary to elucidate whether temporal differences exist in the rate of recovery between the sexes, a current limitation of the study. Such investigations should focus on delineating the specific effects of sex differences during the acute phases of VML injury and assess whether hormonal manipulations exert a lasting influence on both the underlying pathology and the efficacy of regenerative treatments.

The most salient finding in this study was that both males and females benefited from MMG treatment. Our findings demonstrated that females treated with autologous muscle grafts showed improvements in muscle function, while males had reduced fibrosis associated with the MMG treatment. Additionally, the increase in intramuscular adipose following MMG treatment, along with the elevated PPARγ expression in females, suggests a potential long-term anti-inflammatory response that warrants further investigation. As our understanding of the complex interplay between biological sex and regenerative treatments advances, future research should focus on earlier time points and the role of sex hormones to refine therapeutic strategies to restore functional recovery following VML injury.

## Methods

### Experimental design

Skeletally mature male (344 ± 8 g) and female (244 ± 16 g) Sprague-Dawley rats (Charles River Laboratories; Wilmington, MA, USA) were subjected to a VML injury in the TA muscle and either received treatment with MMG as a primary intervention or no treatment (i.e., No Repair). Animals were randomly assigned to one of four experimental groups: (1) male without intervention (M-NR; *n* = 18), (2) male with MMG treatment (M-MMG; *n* = 18), (3) female without intervention (F-NR; *n* = 18), or (4) female with MMG treatment (F-MMG; *n* = 18). In vivo neuromuscular functional capacity was assessed in the injured limbs at the study endpoint of 56 days post-VML, based on prior published studies that established chronic functional deficits by this time point^21,24,47–49^. Animals were subsequently euthanized with Euthasol (Virbac AH Inc.; Westlake, TX, USA) delivered via intracardiac injection. TA and EDL muscles of the hindlimbs were harvested for either molecular or histological analysis. All animals were exposed to a 12-hour light/dark cycle and had ad libitum access to food and water. All protocols and animal care guidelines were approved by the Institutional Animal Care and Use Committee at the Uniformed Services University of the Health Sciences (Protocol SUR-22–082; USUHS; Bethesda, MD, USA). All experiments were conducted in compliance with the Animal Welfare Act, the Implementing Animal Welfare Regulations, the principles of the Guide for the Care and Use of Laboratory Animals, and the Animal Research: Reporting of In Vivo Experiments (ARRIVE) guidelines.

### VML injury model

The procedure for creating a unilateral defect was performed based on a well-characterized rodent model of VML in the TA muscle^22,24,50,51^. Briefly, prior to surgery all animals received Ethiqa XR (0.65 mg/kg) containing 72-hour extended release buprenorphine, by subcutaneous injection and maintained a continuous plane of anesthesia by isoflurane (1 − 3%). Using aseptic technique, a lateral incision was created in the TA muscle of the left hindlimb and a full thickness VML defect was created by pressing a 6 mm biopsy punch into the belly of the muscle. For the MMG treatment, autologous muscle defects were minced using sterile surgical scissors and then re-implanted into the VML defect of the TA muscle. Wounds were closed by suturing individual layers (i.e., fascia followed by skin) and a topical antibiotic was applied to the closure. Following surgery, all animals were monitored daily for 3 days to assess complications with wound closure, dehydration, and pain management. All animals recovered from the VML procedure and no adverse events were observed for the duration of the study. Furthermore, no animals required additional analgesia beyond the initial injection.

### In-vivo neuromuscular strength assessments

At the study endpoint of 56 days, isometric torque was measured in vivo in the VML injured hindlimbs of anesthetized rats (isoflurane; 1.5 − 2.0%) using a dual-mode muscle lever system (Model#305 C-LR; Aurora Scientific Inc.; Aurora, Canada). Transcutaneous electrodes were inserted on either side of the common peroneal nerve and the optimal stimulation voltage was determined for each animal with a series of twitch and tetanic contractions (150 Hz, 0.1 ms pulse width, 400 ms train). Then, the synergist muscles were severed (i.e., tenotomy) to capture isolated in vivo torque measurements from the TA muscle^52^. Isometric tetanic torque was measured across a range of frequencies (10–200 Hz) for force-frequency analysis. The procedure was repeated on the contralateral uninjured limb, with only maximum isometric tetanic torque collected at 150 Hz post-tenotomy.

### Protein measurement

Frozen TA muscles collected at 56 days post-injury were crushed into powder and then suspended in T-PER™ (ThermoFisher; Waltham, MA, USA) lysis buffer and Halt™ Protease Inhibitor Cocktail 100X (Thermo Fisher Scientific Inc.; Waltham, MA, USA) at a concentration of 100 mg/mL. The suspensions were homogenized and centrifuged at 10,000xg for 5 min at 4 °C. The supernatant was collected, and total protein concentration was quantified using a Pierce™ BCA Protein Assay Kit (Thermo Fisher Scientific Inc.; Waltham, MA, USA). Proteins were measured from muscle lysate using ELISA kits (MyBiosource Inc; San Diego, CA, USA or Novus Biologicals; Centennial, CO, USA) for the following targets: peroxisome proliferator-activated receptor gamma (PPARƔ), collagen type 1 alpha 1 (COL1A1), myogenin, myosin heavy chain 1 (MyHC-2x), myosin heavy chain 2 (MyHC-2a), myosin heavy chain 3 (MyHC-embryonic), myosin heavy chain 4 (MyHC-2b), myosin heavy chain 8 (MyHC-neonatal), synapsin-1, neural cell adhesion molecule-1 (NCAM-1), and tubulin β3. ELISA plates were measured at specific wavelengths using an Infinite M200 Pro spectrophotometer (Tecan; Männedorf, Switzerland) and analyzed according to manufacturer instructions. Protein concentrations were calculated by interpolation from the generated standard curve and normalized to total protein concentration quantified from the BCA assay. Protein levels were evaluated at a sample size of 8 to 9 muscles per group until sample availability was depleted, then 3 to 5 muscles per group were evaluated.

### Histology

TA muscles collected at the study endpoint of 56 days were formalin fixed (Sigma-Aldrich; St. Louis, MO, USA) and paraffin embedded for histological staining. Tissues were sectioned at a thickness of 5 μm. Sections stained with Picrosirius Red (PSR; Abcam; Cambridge, United Kingdom) or Hematoxylin and Eosin (H&E; Manufacture Information) followed the suggested manufacturer protocol and then sealed with Surgipath Micromounting Medium (Leica; Wetzlar, Germany). For sections stained with Wheat Germ Agglutinin-AF488 (WGA; Thermo Fisher Scientific Inc.; Waltham, MA, USA), tissues were incubated with WGA (1:40), then sealed with VECTASHIELD Vibrance^®^ Antifade Mounting Medium (Vector Laboratories; Newark, CA, USA). Tissues were imaged at 10X magnification for PSR, H&E, and WGA with an Axio Scan Z1 (Zeiss; Oberkochen, Germany) slide scanning microscope and acquisition parameters were standardized for all samples.

### Collagen quantification

Collagen content was measured from muscle cross-sections taken at the defect region and quantified as a percentage of total tissue area per sample. Images were processed in ImageJ software v.1.54f (http://imagej.org) and collagen and total tissue area parameters were isolated by color using the Color Deconvolution v.3.0.3 (https://imagej.net/plugins/colour-deconvolution) and the band-pass filter Threshold Colour v.1.16 (https://imagejdocu.list.lu/plugin/color/threshold_colour/start) plug-ins^53–55^. Images were then binarized using a global threshold. Tissue area measurements were calculated from the cross-section of muscle samples in the binarized image. The percentage of collagen per total tissue area was then calculated for each TA muscle. A sample size of *n* = 5–7 muscle samples was analyzed for each group.

### Myofiber analysis

Myofiber metrics were analyzed from VML injured and uninjured muscle cross-sections and quantified for minimum Feret diameter, total fiber count, and myofiber distribution. Images were processed using ImageJ software v.1.54f (http://imagej.org) and Myosoft v.15 (https://github.com/Hyojung-Choo/Myosoft) plugins^56^. Briefly, the WGA channel of each image was subjected to a threshold and binarized to isolate muscle fibers using the Trainable Weka Segmentation^57^. The black and white images were then analyzed in ImageJ for myofiber metrics and the raw data files were imported into R software v.4.2.1 (https://www.r-project.org/) for final analysis^58^. A sample size of *n* = 4–5 muscle samples was analyzed for each group.

### Adipose quantification

Adipose measurements were calculated from H&E stained cross-sections in the defect region per muscle and quantified as a percentage of VML defect area per sample. Images were processed in ImageJ software v.1.54f (http://imagej.org) where adipose and total tissue area parameters were isolated by color using the Color Deconvolution v.3.0.3 (https://imagej.net/plugins/colour-deconvolution) and the band-pass filter Threshold Colour v.1.16 (https://imagejdocu.list.lu/plugin/color/threshold_colour/start) plug-ins. Images were then subjected to a global threshold. For VML defect area calculation, an additional processing step was performed to fill in gaps within the muscle sample to measure the tissue cross-section in the binarized image. The percentage of adipose per area was then calculated for each TA muscle. A sample size of *n* = 4–7 muscle samples was analyzed for each group.

### Statistical analysis

All experiments were performed to include at least three biological replicates. Number of muscles analyzed for each experiment is listed in the figure legends. All data are presented as mean ± standard deviation (SD) unless stated otherwise. Data were analyzed using GraphPad Prism software v.9.3.1 (https://www.graphpad.com; GraphPad San Diego, CA, USA) and evaluated for normal distributions and equal variance prior to analysis. Dependent variables passed assumptions for two-way analysis of variance (ANOVA). Mixed-effect models were used if any values were missing from the dataset. In the event of a significant interaction or main effect of treatment or sex, a Sidak’s post-hoc comparison test was performed. Additionally, a two-sided Kruskal-Wallis test was used for evaluation of equal distributions. Statistical significance was achieved at an alpha of 0.05.

## Electronic supplementary material

Below is the link to the electronic supplementary material.

Supplementary Material 1

## Data Availability

The datasets used and/or analyzed during the current study are available from the corresponding authors upon a reasonable request.
