# Supplementary material for: Evaluation of biological sex on endstage pathobiology and regenerative treatment of volumetric muscle loss
Source: Sci Rep. 2025 Jul 1;15:21399. doi: 10.1038/s41598-025-05166-y (PMC12215837; doi:10.1038/s41598-025-05166-y)
Supplement: Supplementary file 1 — Supplementary Material 1 [file 41598_2025_5166_MOESM1_ESM.docx]

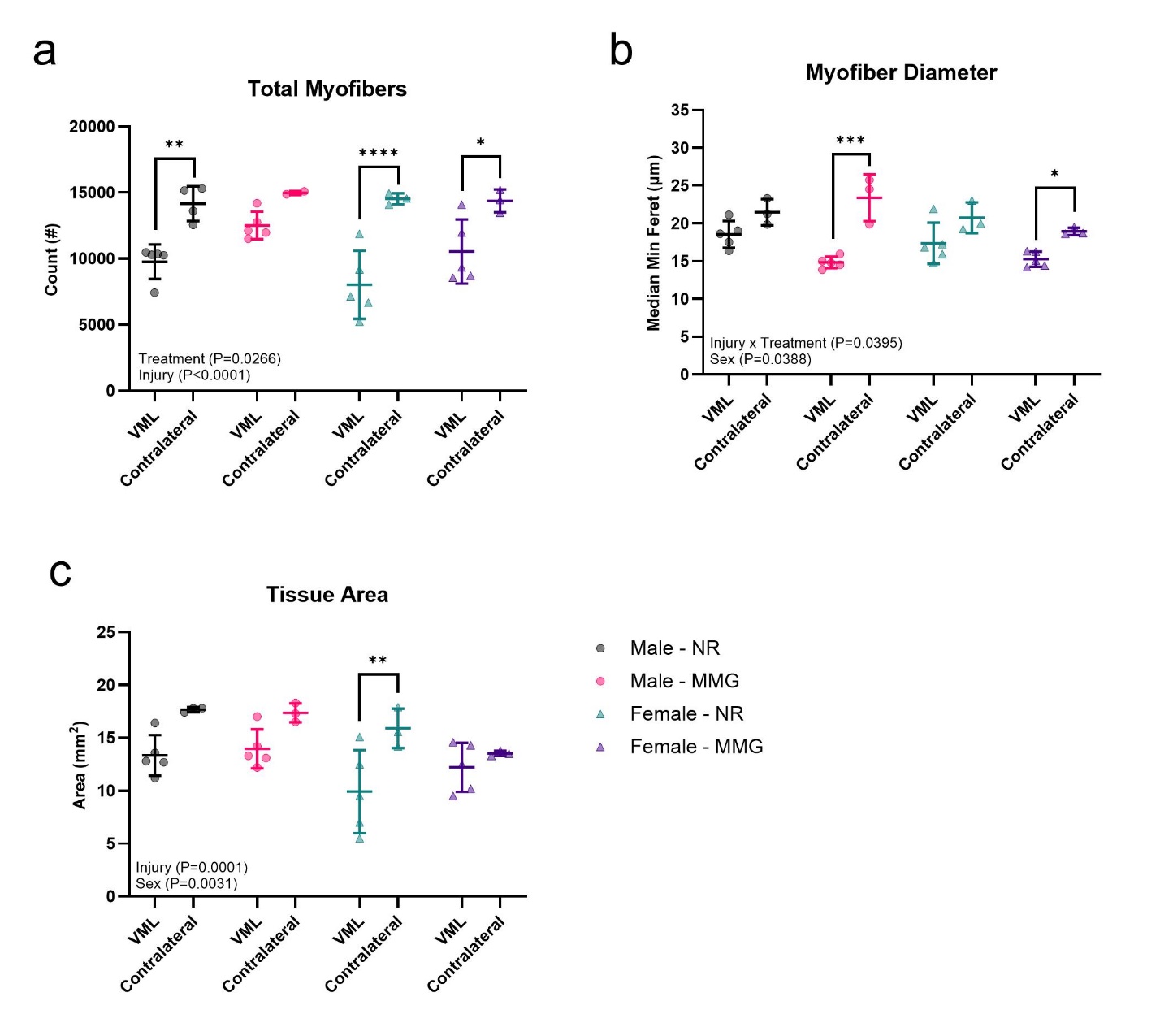


**Supplementary Fig. S1. Comparison of muscle regeneration outcomes in VML-injured and uninjured contralateral limbs.** Total muscle fiber count (**a**), median myofiber diameter (**b**), and tissue area (**c**) were quantified to assess the effects of biological sex and regenerative treatment on muscle regeneration in VML (*n* = 5 muscles/group) compared to uninjured contralateral (n = 3 muscles/group) muscles. Untreated males, MMG-treated males, untreated females, and MMG-treated females are represented by black, pink, teal, and purple markers, respectively. Data are presented as mean ± SD. Statistical significance is indicated by *(P<0.05), **(P<0.01), ***(P<0.001), and ****(P<0.0001) assessed using two-way ANOVA with Sidak’s post hoc test.
